# Effects of Telenursing on Self-Care and Healthcare Outcomes in Patients with Heart Failure: A Systematic Review

**DOI:** 10.3390/clinpract16100183

**Published:** 2026-09-30

**Authors:** Rita Costa, Sara Pires, Idalina Gomes, José Moreira, Elisabete Alves, César Fonseca, Ana Ramos

**Affiliations:** 1 Local Health Unit of São José, 1150-199 Lisbon, Portugal; ritamarquescosta@campus.enfermagem.ulisboa.pt; 2 Nursing Research Innovation and Development Centre of Lisbon (CIDNUR), School of Nursing, Universidade de Lisboa, 1600-190 Lisbon, Portugal; sarapires@enfermagem.ulisboa.pt (S.P.); idgomes@enfermagem.ulisboa.pt (I.G.); 3 Comprehensive Health Research Centre (CHRC), 7004-516 Évora, Portugal; jose.moreira@uevora.pt (J.M.); elisabete.alves@uevora.pt (E.A.); cfonseca@uevora.pt (C.F.); 4 Escola Superior de Enfermagem São João de Deus, University of Évora, 7004-516 Évora, Portugal

**Keywords:** telenursing, self-care, outcome assessment, heart failure

## Abstract

**Background/Objectives:** Heart failure (HF) remains a major public health challenge, requiring continuous management and high patient adherence to prevent exacerbations. Telenursing offers a scalable, tailored solution for remote monitoring and education; however, evidence regarding optimal individualized intervention models remains heterogeneous, necessitating a systematic synthesis within the framework of precision care. This study aims to identify the effects of telenursing on self-care capacity and health outcomes in patients with heart failure. **Methods:** A systematic review was conducted following JBI methodology and PRISMA guidelines. Six databases (MEDLINE, Cochrane, CINAHL, Scopus, Web of Science, Google Scholar) were searched for studies (2020–2025). Quality was assessed using JBI Critical Appraisal Tools (threshold > 60%). **Results:** Fifteen studies were included from the 748 identified. Three core personalized modalities were categorized: tailored self-care digital programs, mobile applications, and integrated telemonitoring systems. Interventions featuring individualized, risk-stratified, and frequent contact demonstrated a significant positive impact on self-care behaviors, NYHA classification, and hospitalization rates. Key components included personalized vital sign alerts, structured virtual interviews, and digital coaching. Nurse-led oversight directly supported clinical responsiveness and medication adherence, though therapeutic efficacy varied according to baseline patient digital literacy and cognitive function. **Conclusions:** Technology-enabled personalized nursing interventions are highly effective in enhancing self-care skills, health literacy, and clinical stability. Future research should prioritize large-scale trials with prolonged follow-up and cost-effectiveness analyses to support multidisciplinary integration.

## 1. Introduction

The global population is aging. It is estimated that the number of people aged 60 years and older will double by 2050 and more than triple by 2100 [1]. In Europe, approximately 25% of the population is aged 60 years or older [2].

Population aging has contributed to the increased prevalence of chronic diseases, and cardiovascular diseases remain the leading cause of mortality worldwide. Elevated blood pressure is the main metabolic risk factor for cardiovascular mortality [3]. Among chronic cardiovascular conditions, heart failure (HF) is defined as a chronic condition in which the heart muscle is unable to pump sufficient blood to meet the body’s metabolic demands [4]. Globally, approximately 64.3 million people are living with HF. In developed countries, prevalence is estimated at around 2%, with an annual incidence of 5 to 10 cases per 1000 individuals. Among adults aged 65 years and older, prevalence increases to approximately 11.8% [5]. Chronic heart failure is the leading cause of hospitalization among older adults, with 24% of patients readmitted within 30 days and approximately 50% rehospitalized within six months [6].

Clinically, HF is classified into four phenotypes based on Left Ventricular Ejection Fraction (LVEF): HF with reduced ejection fraction (LVEF ≤ 40%), mildly reduced ejection fraction (LVEF 41–49%), preserved ejection fraction (LVEF ≥ 50%), and improved ejection fraction (reflecting a baseline LVEF ≤ 40% that subsequently increases by at least 10 points to >40%) [7]. To evaluate symptoms and functional capacity, patients are categorized using the New York Heart Association (NYHA) classification on a scale from Class I (no limitation of physical activity) to Class IV (symptoms present even at rest) [8]. The symptom burden experienced by patients with Heart Failure is complex and multifactorial. The primary manifestations, which are most strongly associated with HF-related hospitalizations, are driven by fluid overload, most notably pulmonary edema, dyspnea (shortness of breath), and peripheral edema. Furthermore, the impact of the disease extends to a profound systemic and psychosocial component, with patients frequently reporting debilitating symptoms such as fatigue, chest and general pain, alongside significant levels of anxiety and depression [9,10]. The daily demands of HF self-care frequently exceed the patient’s individual capacity, creating a critical deficit that necessitates targeted and continuous healthcare interventions [11,12]. These limitations in the capacity for self-care can be influenced by health literacy, cognitive and functional decline, the burden of comorbidities, and constraints related to time and environmental resources [13,14].

To improve access to care, continuity, and proximity between individuals and nursing services, telehealth expanded significantly during the COVID-19 pandemic [15]. Telenursing is an innovative paradigm in digital healthcare, defined as the use of information and communication technologies to deliver nursing care from a distance [16]. Situated within the broader domain of eHealth, the overarching application of digital technologies and information systems to healthcare delivery, telenursing encompasses a broad spectrum of activities, ranging from telephone counseling to complex home monitoring via remote devices, effectively transcending spatial limitations [17]. With the technological evolution of mobile health (mHealth) applications, wearable biosensors, and wireless peripherals, telenursing interventions have increasingly leveraged mHealth platforms to enable continuous, real-time data transmission and interactive patient engagement outside traditional clinical settings [18,19]. Telemonitoring is described as a specialized model of care within this eHealth and mHealth framework that involves remote monitoring of biometric parameters, enabling early detection of HF deterioration, improved clinical classification, and timely implementation of preventive interventions [20]. By enabling continuous and coordinated care across different levels of the health system, telenursing allows for prompt, needs-based interventions, positioning itself as a pioneering and rapidly growing delivery system for the modern age [21,22].

Within the European Union, digital health is recognized as a key instrument to improve the efficiency and quality of care, expand access, empower individuals in the management of their health, and support healthcare professionals [23]. Global investment in digital health is supported by the Global Strategy on Digital Health 2020–2025, which aims to strengthen health systems and empower communities in self-care [24]. The justification for this study lies in the critical gap between nurses’ positive attitudes toward telenursing and their currently insufficient telehealth knowledge in cardiac care [25]. Despite the growing evidence on digital health in heart failure, previous systematic reviews have largely focused on macro-clinical endpoints such as mortality and readmission rates [26], overall quality of life [27], or broad technology adoption driven by the COVID-19 pandemic [28]. Consequently, a critical gap remains in synthesizing how structured telenursing—as an active, professional nursing model encompassing direct clinical symptom triage, interactive educational coaching, and structured virtual follow-ups—directly influences daily self-care capacity and specific health outcomes. To bridge this gap and position telenursing as a sustainable chronic care model, this review aims to identify the effects of telenursing on self-care capacity and health outcomes in patients with heart failure.

## 2. Materials and Methods

This systematic literature review was conducted following the Joanna Briggs Institute (JBI) methodology [29]. The search strategy and article analysis were carried out in accordance with the PRISMA guidelines for systematic reviews and meta-analysis extensions [30] (see Supplementary Materials). The review protocol was registered on the PROSPERO (CRD420251080142) platform to avoid duplication of scientific evidence.

### 2.1. Eligibility Criteria

The inclusion and exclusion criteria were established a priori based on the Population, Intervention, Comparison, and Outcome (PICO) framework [31], adhering to the guiding principles for systematic reviews. Accordingly, the primary research question was formulated as follows: What are the effects of telenursing interventions (I), compared to standard care, non-digital care, or baseline parameters (C), on self-care capacity and health outcomes (O) among adult patients with heart failure (P)?

The PICO elements were defined as:

(i) Population (P): Adult patients (≥18 years of age) with diagnosed Heart Failure (HF).
(ii) Intervention (I): Telenursing interventions (e.g., nurse-led telemonitoring, mHealth applications, structured virtual coaching, remote symptom triage).
(iii) Comparison (C): Standard care or non-digital nursing care.
(iv) Outcome (O): Primary outcomes included self-care capacity. Secondary outcomes included health-related parameters (NYHA functional class, 30-day readmissions, medication adherence, and health literacy).

Eligible study designs included randomized controlled trials (RCTs), quasi-experimental studies, cohort studies, and analytical cross-sectional studies. For comparative studies (RCTs and quasi-experimental trials), intervention effects were evaluated by extracting available effect estimates (e.g., Mean Differences [MD], Standardized Mean Differences [SMDs], or Odds Ratios [ORs] with 95% Confidence Intervals). Single-group or observational studies were synthesized narratively to provide implementation data.

The search was restricted to articles published in English, Portuguese, and Spanish. Crucially, the search was limited to articles published within the last five years (2020–2025). This temporal restriction is methodologically justified by the rapid evolution and obsolescence of digital health technologies, as well as the unprecedented paradigm shift in telehealth adoption and remote care delivery catalyzed by the COVID-19 pandemic. Including older studies could introduce bias by analyzing obsolete technological frameworks that no longer reflect current clinical practices.

Studies were excluded if they did not explicitly address the research question, lacked a focus on nursing-led care, or did not present clearly defined empirical objectives. Furthermore, non-primary research articles, such as previous literature reviews, editorials, commentaries, and opinion pieces, were excluded to maintain the empirical rigor of the synthesized evidence.

### 2.2. Search Strategy

To ensure the originality of this systematic review, a preliminary search was conducted, confirming the absence of previously published reviews or registered protocols on this specific topic. For the purpose of this review, telenursing was operationalized as any remote, technology-mediated care delivery, symptom triage, educational coaching, or clinical monitoring performed by or under the direct coordination of registered nurses [16,32].

A literature search was conceptualized and executed by two independent investigators (R.C. and A.R.) and validated by a third author. The search was performed across five major electronic databases: MEDLINE (via PubMed), Cochrane Library, CINAHL Complete, Scopus, and Web of Science. Broad, high-sensitivity search terms were deliberately combined to capture diverse nurse-led digital interventions: “telenursing”, “nursing care”, “patient participation”, “telemedicine”, “digital health”. To complement electronic database searches and reduce publication bias, manual forward and backward citation tracking of all included studies and relevant systematic reviews was performed. Concurrently, grey literature was explored via Google Scholar, while the primary trial registry ClinicalTrials.gov was screened independently to identify completed unpublished studies or relevant ongoing trials. The search period was restricted from 1 January 2020 to 24 June 2025. This timeframe was explicitly selected to isolate contemporary post-2020 digital nursing delivery models, capturing the structural paradigm shift, updated clinical guidelines, and standardized remote care workflows established following the onset of the COVID-19 pandemic.

The search syntax tailored for each database is detailed in Table 1.

### 2.3. Evaluation of Risk of Bias and Methodological Quality of Studies

To maintain methodological rigor and minimize potential risk of bias, two researchers independently screened and evaluated the full text of all eligible articles. Methodological quality and certainty of evidence were critically appraised using design-specific Joanna Briggs Institute (JBI) critical appraisal tools [29]. For randomized controlled trials, the 13-item JBI tool was used to evaluate domains such as the randomization process, allocation concealment, baseline group comparability, blinding, consistency of outcome measurement, and follow-up completeness [33]. Quasi-experimental studies were appraised using the 9-item JBI checklist, focusing on causal relationships, group comparability, control of confounding variables, and outcome reliability [34]. Observational evidence was assessed using the 11-item JBI tool for cohort studies (evaluating exposure measurement, confounding management, and follow-up) [35] and the 8-item JBI tool for analytical cross-sectional studies (focusing on inclusion criteria clarity, setting descriptions, exposure/outcome validity, and confounding management) [36]. Each item across the appraisal tools was scored as “yes” (1 point), “no” (0 points), “unclear” (0 points), or “not applicable”. Each item across the appraisal tools was scored as “yes” (1 point), “no” (0 points), “unclear” (0 points), or “not applicable”. The overall score was calculated based on the proportion of fulfilled criteria (X/Y), allowing classification into quality tiers (high quality: >80%; moderate quality: 70–80%; low quality: 60–70%) to provide a standardized overview of methodological strength across study designs [12]. Evaluators resolved discrepancies through consensus with a third reviewer.

We managed the screening and selection process using Rayyan software (Cambridge, MA, USA; 2024, https://www.rayyan.ai/, accessed on 2 April 2026). The literature was sequentially evaluated through title and abstract screening, followed by a full-text review of eligible reports against pre-established inclusion criteria. Table 2 summarizes the methodological quality assessment across all included studies.

### 2.4. Data Extraction and Synthesis

Data extraction was performed independently by two investigators (R.C. and A.R.) using a standardized electronic form to ensure consistency and minimize selection bias. Any discrepancies between the reviewers during this stage were resolved through consensus or by consulting a third senior investigator to maintain inter-rater reliability. Data synthesis was organized in six parts: study references (author, year, and country), main aim, study design, sample characteristics (sample size and average age), measure instruments, characteristics of telenursing intervention program (components and delivery time), and clinical outcomes.

### 2.5. AI Use Statement

During the preparation of this manuscript, the authors used Gemini 1.5 and NotebookLM (Google) (https://sites.google.com/view/notebook-lm?pli=1&authuser=0, accessed on 2 April 2026) for the purposes of text refinement and graphic generation. The authors have reviewed and edited the output and take full responsibility for the content of this publication.

## 3. Results

### 3.1. Study Selection

**Figure 1:**
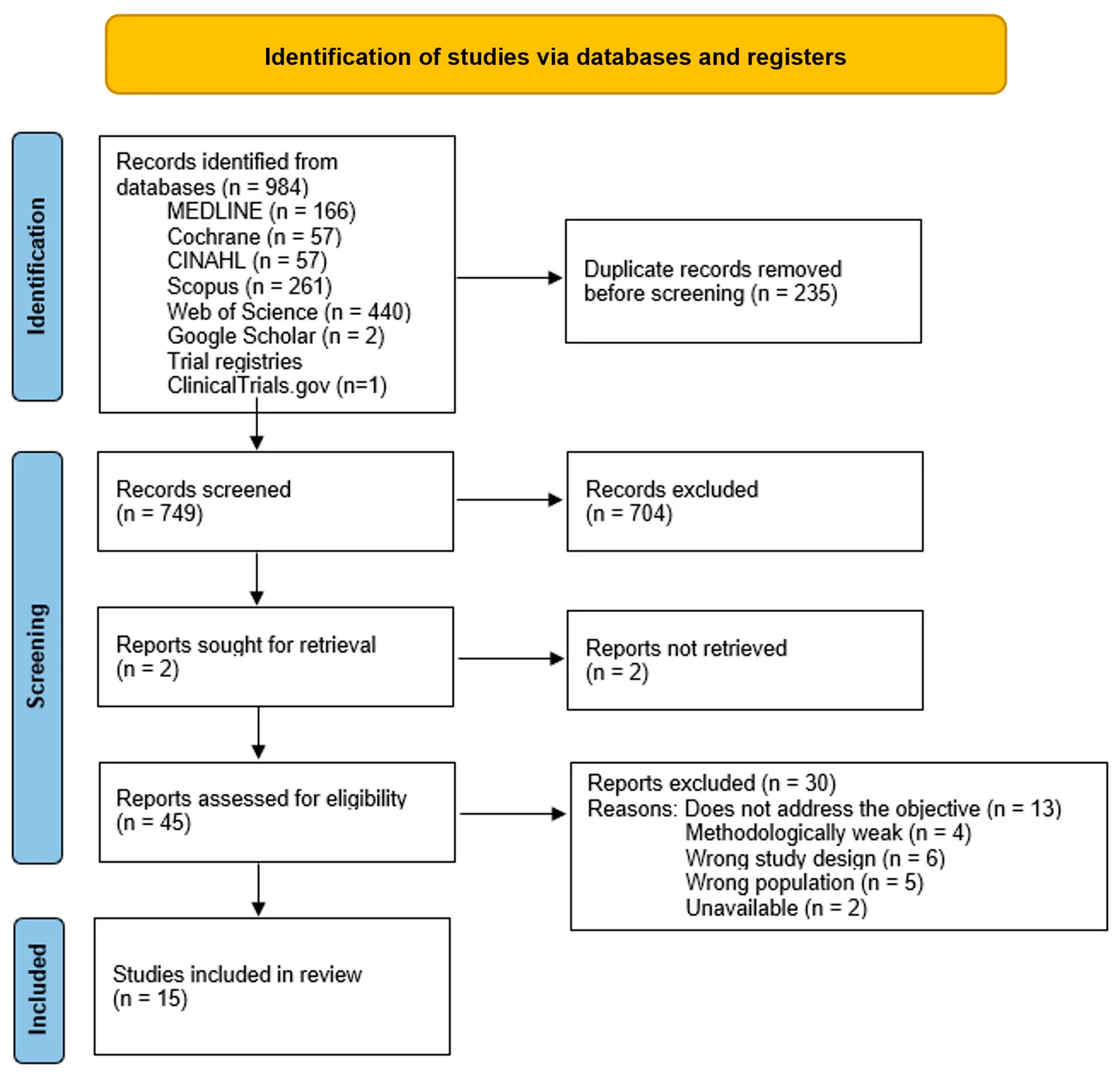
PRISMA flowchart of the article and publication selection process.

The initial search identified a total of 984 records. After the removal of duplicates (*n* = 235), 749 records remained. Screening of titles and abstracts led to the exclusion of 704 records, resulting in 45 potentially eligible studies. Following full-text assessment, 30 articles were excluded. The PRISMA flow diagram illustrating the study selection process is presented in Figure 1. A total of 15 studies were included in the final analysis.

### 3.2. Study Characteristics

A total of 15 studies met the eligibility criteria and were included in this systematic review, as detailed in Table 3 and Table 4. The evidence synthesis reveals a global interest in telenursing for heart failure (HF), with studies spanning four continents: Asia (*n* = 5: Korea, Japan), North America (*n* = 5: USA, Canada), Europe (*n* = 4: Finland, Germany, Italy, United Kingdom), and South America (*n* = 1: Argentina).

Regarding study design, the body of evidence is characterized by methodological diversity and is distributed as follows: quasi-experimental studies (*n* = 7); randomized controlled trials (RCTs) (*n* = 6); one analytical cross-sectional study (*n* = 1); and one retrospective case–control study (*n* = 1).

The cumulative sample size across all studies was 6660 participants, though it is important to note that a single large-scale retrospective study accounted for 6065 of these individuals [50]. Excluding this outlier, the median sample size for primary clinical trials was 41 participants (range: 12 to 101). The population consisted primarily of older adults, with mean ages ranging from 52 to 72.4 years. While most studies targeted the patients exclusively, three studies specifically adopted a dyadic approach, incorporating caregivers into the telenursing intervention to evaluate collaborative self-care. Clinically, the majority of participants were classified within NYHA Classes II and III, reflecting a symptomatic population with a significant need for intensive self-management support.

## 4. Discussion

The critical analysis of the results from this systematic review is structured around four fundamental axes that provide a holistic understanding of the effects of telenursing on HF self-care, as well as the facilitating factors and barriers to implementation.

### 4.1. Architecture and Delivery of Personalized Telenursing Interventions

The technological landscape of telenursing for Heart Failure (HF) is characterized by a diverse operational architecture, categorized into three functional modalities. Self-care digital programs represent the most frequent approach, emphasizing structured education and behavioral coaching [38,43,44,45,47,49,50].

These programs often utilize a combination of virtual sessions and manualized support. For example, the REACH-HF program [47] provided patients with an HF manual, exercise and stress management plans, and a progress tracker, complemented by family support materials. Similarly, the CASE-CCT intervention integrated computerized cognitive training (CCT), in which patients navigated complex virtual environments to achieve goals, alongside formal educational sessions [43].

The second modality consists of mobile application-based strategies that prioritize patient-led data entry and asynchronous communication [37,39,42,48]. These interventions often feature personalized components: some utilized a chatbot delivering messages four times weekly [48,49], while others developed an app offering diary monitoring, reminders, and chat support directly guided by cardiac nurses [45]. The third modality, integrated telemonitoring systems, focuses on continuous physiological surveillance [41,44,45,50,51]. These systems typically employ hardware such as digital scales, blood pressure monitors, and Android tablets [51] or voice-activated assistants like Alexa [40] to collect and sync data to cloud-based platforms for nursing review.

The materials utilized across these studies were highly multidimensional. Beyond digital interfaces, several programs incorporated physical and educational resources to ensure a hybrid approach. The study conducted by Longhini et al. [45] offered a choice between mobile apps and paper materials to accommodate different literacy levels. Interventions frequently included personalized SMS [38], video consultations [39], and structured telephone interviews [43,50]. A recurring component was the automated alert system, which triggered nurse notification upon detecting weight gain or symptom exacerbation [41,42,50]. This “human-in-the-loop” feedback loop, where nurses review alerts and provide tailored instructions, remains the decisive factor for success [39].

The temporal scale of the included interventions reflects a transition from acute support to long-term chronic management. The duration of telenursing programs ranged from 8 weeks [38,49] to a maximum of 12 months [44,50]. A significant cluster of studies utilized a 3-month (12-week) monitoring period, which was considered the threshold for observing behavioral stabilization in self-care [37,39,40,41,46]. The dosage or frequency of contact also varied: while telemonitoring was typically a daily habit [37,41,50], educational or coaching sessions were delivered in structured blocks, such as 10 sessions [43] or an average of 5 facilitator sessions totaling nearly 5 h of contact [47]. This variability suggests that while monitoring is continuous, the coaching element is often front-loaded to empower the patient during the early stages of the program.

### 4.2. Effects of Telenursing on Self-Care Profile and Health Outcomes

The synthesis of available evidence underscores the critical role of telenursing interventions in optimizing the clinical status and behavioral profiles of patients with heart failure. The primary efficacy of these digital health models lies in their impact on self-care behaviors, with ten out of eleven evaluating studies reporting statistically significant positive effects [38,40,41,42,43,45,47,48,49,51]. The single investigation reporting non-significant outcomes in this domain [37] evaluated a substantially older cohort (mean age: 70.3 years) characterized by lower baseline educational attainment, suggesting that advanced age and limited digital literacy may attenuate the translation of digital content into sustained behavioral modification within a 3-month trial period.

Improvements in self-care behaviors are structurally supported by parallel gains in health literacy and disease-specific knowledge [45,48,49]. However, one study [42] observed no significant post-intervention changes in knowledge scores, a finding likely attributable to ceiling effects, as 58% of participants engaged in daily self-monitoring prior to enrollment. Similarly, while telenursing interventions consistently enhanced electronic health literacy [48,49], these benefits did not extend to psychosocial outcomes such as depressive symptoms or caregiver burden, indicating that digital health tools alone are insufficient for addressing complex emotional and psychological needs [48].

Enhanced self-care directly translated into objective clinical improvements. All trials assessing functional capacity via the New York Heart Association classification demonstrated statistically significant favorable shifts [37,39,44]. Furthermore, Health-Related Quality of Life scores improved across most cohorts [39,46,47,48]. The absence of significant quality-of-life gains in specific trials [37,42] may be explained by modest sample sizes and brief follow-up intervals (2 to 3 months), which lack adequate duration to capture longitudinal changes in broad health-related quality of life constructs.

Regarding healthcare utilization, four out of five studies concluded that telenursing effectively attenuated acute care episodes and hospital readmission rates [39,41,48,50]. Notably, Knoll et al. [50] established significant reductions in mortality and total hospital days through integrated telemonitoring combined with structured nurse coaching. Conversely, Shara et al. [40] observed an unintended increase in emergency department presentations and hospital admissions. This paradoxical outcome suggests that while automated voice interfaces successfully augmented symptom awareness, the absence of a structured, nurse-led triage protocol to filter incoming telemetry data precipitated premature acute care utilization. This finding emphasizes that technological infrastructure must be paired with clinical decision-making and a therapeutic nurse–patient relationship to manage stability safely without overburdening acute healthcare systems.

From a biostatistical perspective, the literature exhibits substantial variation in study design, sample size, and analytical power. A subset of small-scale pilot trials and feasibility studies—including Arslanian-Engoren et al. [43] (*n* = 12), Yokota et al. [42] (*n* = 24), Kitsiou et al. [38] (*n* = 27), and Yanicelli et al. [41] (*n* = 30)—were constrained by small sample sizes, leaving them underpowered to detect differences in secondary hard endpoints such as all-cause mortality or 30-day readmissions. Nevertheless, these investigations demonstrated high feasibility, low attrition, and preliminary efficacy in patient-reported outcome measures, such as self-care maintenance and self-efficacy. Similarly, feasibility trials exploring automated voice interfaces by Shara et al. [40] and remote monitoring protocols by Marier-Tétrault et al. [46] reported reductions in absolute risk for unscheduled healthcare utilization among highly adherent sub-cohorts (adherence > 80%), although several implementation pilots did not report inferential statistics or 95% confidence intervals for these secondary endpoints.

Conversely, large observational cohorts and methodologically rigorous randomized controlled trials provided higher statistical precision and population-level inference. The retrospective cohort study by Knoll et al. [50] (*n* = 6065) utilized high statistical power to demonstrate significant survival advantages across 1-year and 2-year timeframes under both intention-to-treat (Hazard Ratio = 0.62, 95% CI: 0.56 to 0.69) and per-protocol (Hazard Ratio = 0.47, 95% CI: 0.42 to 0.53) analytical models. Methodological reporting quality varied across the literature: while several studies relied primarily on null hypothesis significance testing (*p*-values), Kitsiou et al. [38] and Purcell et al. [47] incorporated standardized effect size metrics (Cohen’s d) and explicit confidence intervals alongside minimum clinically important difference thresholds and formal cost-effectiveness estimates.

Longitudinal studies incorporating multivariate modeling demonstrated distinct mechanisms influencing self-care trajectory and clinical deterioration. Son et al. [48] utilized Generalized Estimating Equations modeling across a 6-month follow-up to confirm independent, sustained improvements in dyadic self-care behaviors (beta = 4.68, *p* = 0.010) and caregiver contributions (beta = 8.76, *p* < 0.001), supporting earlier observations by Longhini et al. [45] regarding dyadic monitoring dynamics. In evaluating digital intervention complexity, Schmaderer et al. [39] established a dose–response pattern, wherein an advanced mobile health intervention with automated risk alerts significantly reduced 90-day hospital readmissions compared with simple mobile health monitoring or usual care (16% vs. 56% vs. 50%, *p* = 0.020). Finally, trials accounting for baseline neurocognitive variables by Choi et al. [37] and Kaneko et al. [44] identified executive dysfunction as an independent risk factor for 12-month heart failure readmission (Adjusted Hazard Ratio = 4.97, *p* = 0.031), illustrating that physiological parameters may improve independently of self-care management changes in populations with cognitive impairment.

### 4.3. Definitions of Health-Related Outcomes and Instruments Measuring Telenursing Outcomes

To establish conceptual clarity and enable cross-study comparison, primary outcomes across the evaluated literature were operationally defined and measured using validated, multidimensional assessment tools. Self-care was operationalized according to the Middle-Range Theory of Self-Care in Chronic Illness, which differentiates between self-care maintenance (routine adherence to treatment and symptom monitoring), self-care perception/monitoring (symptom recognition), and self-care management (autonomous response to clinical deterioration). Behavioral trajectories were primarily evaluated using the Self-Care of Heart Failure Index (SCHFI) [38,43,45,47] and the European Heart Failure Self-care Behavior Scale (EHFScB) [37,41,42,48,49,50]. While the SCHFI assesses maintenance, management, and self-efficacy domains, the EHFScB quantifies the execution frequency of discrete behavioral protocols, including daily weight tracking, dietary sodium and fluid restriction, and pharmacotherapeutic compliance.

Health-Related Quality of Life (HRQoL) was operationally defined as a multidimensional construct encompassing physical limitations, symptom frequency, social functioning, and psychological well-being. Disease-specific HRQoL was measured via the Kansas City Cardiomyopathy Questionnaire (KCCQ-12) [38,46] and the Minnesota Living with Heart Failure Questionnaire (MLHFQ), whereas generic health state utility was captured using the EuroQol 5-Dimension (EQ-5D) index [39,47,48], evaluating mobility, daily activity performance, pain/discomfort, and anxiety/depression. Clinical functional capacity was operationalized using the New York Heart Association (NYHA) functional classification system [37,39,44] as an objective proxy for physical symptom burden during daily exertion. Healthcare utilization was operationally defined and sub-classified into discrete clinical events: all-cause hospital readmissions, heart failure-specific readmissions, unplanned emergency department (ED) presentations, and index hospitalization length of stay (total hospital days).

Given the digital delivery mechanism of the evaluated interventions, study protocols further incorporated specialized psychometric and cognitive instruments to assess digital literacy, engagement, and baseline cognitive capacity. Specifically, digital literacy and software engagement were evaluated using the eHealth Literacy Scale (eHEALS) to measure patient self-efficacy in acquiring, appraising, and applying electronic health information [48,49], alongside the Mobile Application Rating Scale (MARS) to quantify objective usability and engagement metrics of software interfaces [48,49]. Furthermore, to account for critical neurocognitive prerequisites, the Trail Making Test (TMT Parts A and B) was employed to quantify executive function, cognitive flexibility, and visual processing speed [44], thereby identifying the cognitive thresholds required for autonomous self-monitoring and effective interaction with digital health platforms.

### 4.4. Facilitators, Barriers, and Implementation Challenges in Digital Health

The heterogeneity across primary studies reflects critical differences in intervention architecture, patient selection, and organizational integration rather than an inherent limitation of digital care. While early telemedicine trials often yielded neutral results due to passive monitoring and unselected cohorts, the COVID-19 pandemic catalyzed a shift toward structured digital models to preserve care continuity [52]. Modern evidence demonstrates that success depends on active nurse-led monitoring, high intervention intensity, and individualized risk stratification.

The real-world implementation of telenursing interventions in heart failure management is governed by a complex interplay of patient-level, technological, and systemic determinants. Key facilitators include enhanced patient-perceived safety, intuitive interface design, and elevated health motivation [49,51]. The presence of a virtual nurse or dedicated digital contact serves as a vital psychological catalyst for sustained engagement [45]. Furthermore, dyadic care models that actively integrate informal caregivers create essential technical and emotional support structures, significantly bolstering protocol adherence and self-care maintenance [47,48].

Conversely, substantial patient-level barriers continue to limit intervention scalability and effectiveness. Limited eHealth literacy and a persistent preference for traditional paper-based self-monitoring tools remain major obstacles, particularly among older populations [45,46]. Baseline clinical vulnerabilities—including executive dysfunction, frailty, advanced age, and lower educational attainment—substantially hinder a patient’s capacity to navigate multi-step digital interfaces and translate telemetry outputs into timely clinical actions [44,45]. These factors raise critical health equity concerns, as socioeconomically disadvantaged or digitally underserved sub-cohorts bear a disproportionate burden of heart failure morbidity yet risk exclusion from non-tailored digital health models, inadvertently widening existing care disparities.

Beyond individual-level barriers, systemic, organizational, and methodological challenges heavily influence intervention outcomes. A primary methodological limitation across the literature is the presence of confounding and unisolated co-interventions. Reported clinical improvements are frequently confounded by concurrent guideline-directed medical therapy titration, multidisciplinary team access, and protocolized specialist follow-ups, making it difficult to isolate the independent effect of digital platforms. Systemically, technical instability, connectivity failures, and a lack of Electronic Health Record interoperability disrupt clinical continuity [46,51]. Simultaneously, continuous remote monitoring introduces significant data privacy risks and governance challenges regarding data transmission security and liability for unreviewed telemetry alerts. From an operational perspective, unfiltered, high-volume data streams generate severe nurse alert fatigue, clinical workflow disruptions, and administrative burden, demonstrating that remote monitoring can paradoxically increase healthcare provider strain if not supported by structured triage protocols.

To overcome these implementation barriers, non-invasive telenursing must be conceptualized within the broader, rapidly evolving landscape of heart failure telemonitoring technologies. As highlighted by Pierucci [53], heart failure telemonitoring spans a wide continuum, ranging from non-invasive mobile software applications to advanced multi-parametric predictive algorithms and implantable hemodynamic sensors, such as CardioMEMS (Abbott Park, Illinois, EUA), HeartLogic (Massachusetts, EUA), and SELENE-HF (Berlin, Germany). While non-invasive telenursing primarily reinforces behavioral self-care, symptom recognition, and patient engagement, automated predictive algorithms and invasive sensors offer subclinical lead times that detect hemodynamic congestion weeks before overt clinical decompensation. Future implementation strategies must therefore leverage a synergistic framework: using multi-parametric diagnostic algorithms to filter false-positive alerts and reduce nursing workload, while preserving nurse-led therapeutic relationships to translate automated telemetry into safe, individualized, and patient-centered clinical care.

Furthermore, future randomized controlled trials employing multi-arm or factorial designs are warranted to dissect the specific active ingredients of telenursing interventions, isolating the relative contribution of nurse contact frequency, remote biometric monitoring, structured education, and behavioral coaching to establish which specific components drive optimal patient outcomes, as highlighted by emerging protocolized frameworks and long-term registry-based clinical trials (ClinicalTrials.gov Identifier: NCT06517836) [54].

### 4.5. Limitations

Despite the systematic approach, this review has limitations that must be acknowledged. First, the methodological quality of the primary studies is constrained by small sample sizes, specifically within early-phase feasibility trials that lacked statistical power to evaluate secondary clinical endpoints, including all-cause mortality and 30-day readmission rates. Furthermore, the unblinded design inherent to digital behavioral and nursing interventions prevented participant and provider masking, introducing potential selection, performance, and detection biases. Search strategies restricted by language criteria may have excluded additional relevant global datasets.

Second, the body of evidence demonstrates substantial methodological, interventional, and clinical heterogeneity, which precluded pooled quantitative meta-analysis. Included investigations varied in study design (ranging from pilot randomized controlled trials to retrospective observational cohorts), follow-up duration (ranging from 30 days to 24 months), and technological architecture (ranging from automated interactive voice response systems to multi-component mobile applications). Interventions frequently co-occurred with unisolated co-interventions, including protocolized guideline-directed medical therapy titration, multidisciplinary heart failure team access, and scheduled clinical contacts, limiting the isolation of the independent treatment effect of the telenursing component.

Finally, operational definitions and psychometric measurement instruments varied across trials, particularly within self-care maintenance subscales and health-related quality of life domains, where point estimates were reported without consistent standard deviations, standardized effect sizes, or explicit 95% confidence intervals. Population characteristics—ranging from younger, digitally literate individuals to frail, older cohorts with documented executive dysfunction—limit the generalizability of these findings across the overall heart failure population.

## 5. Conclusions

This systematic review demonstrates that telenursing interventions are a versatile and effective strategy for improving the clinical and behavioral profile of individuals with Heart Failure. The evidence consistently shows that nurse-led digital programs, mobile applications, and telemonitoring systems significantly enhance self-care behavior, health awareness, and eHealth literacy. Furthermore, these interventions have a measurable impact on functional status, as evidenced by improvements in NYHA classification, and play a substantial role in reducing hospitalization rates and enhancing Health-Related Quality of Life.

A key finding of this review is that the success of telenursing is not merely dependent on the technology itself, but on the nurse–patient therapeutic partnership. Providing personalized feedback and clinical triage is essential to prevent the over-utilization of health services and to ensure patient safety. While barriers such as low digital literacy, advanced age, and executive dysfunction persist, they can be mitigated through tailored interventions and the involvement of the patient–caregiver dyad.

For clinical practice, these results suggest that telenursing should be integrated into routine follow-up protocols for HF, particularly for high-risk populations post-discharge. Future research should focus on long-term longitudinal studies (beyond 12 months) and on the standardization of intervention dosages to determine the optimal frequency of contact. In conclusion, telenursing represents a critical evolution in cardiac care, optimizing resources and self-care of the patients to manage their health safely in their home environment.

## Figures and Tables

**Table 1:** Search strategy in the database.

Search	MEDLINE and Cochrane
1	“telenursing” OR “nursing care” OR “patient participation” OR “telemedicine” OR “digital health”
2	“self-care”
3	“heart failure” OR “heart failure, diastolic” OR “heart failure, systolic”
4	(“telenursing” OR “nursing care” OR “patient participation” OR “telemedicine” OR “digital health”) AND “self-care” AND (“heart failure” OR “heart failure, diastolic” OR “heart failure, systolic”)
Search	CINAHL Complete
1	“telenursing” OR “nursing care” OR “patient participation” OR “telemedicine” OR “digital health”
2	“self-care”
3	“heart failure” OR “heart failure nursing”
4	(“telenursing” OR “nursing care” OR “patient participation” OR “telemedicine” OR “digital health”) AND “self-care” AND (“heart failure” OR “heart failure nursing”)
Search	Scopus
1	TITLE-ABS-KEY(“telenursing” OR “nursing care” OR “patient participation” OR “telemedicine” OR “digital health”)
2	TITLE-ABS-KEY(“self-care”)
3	TITLE-ABS-KEY(“heart failure” OR “heart failure, diastolic” OR “heart failure, systolic”)
4	(TITLE-ABS-KEY(“telenursing” OR “nursing care” OR “patient participation” OR “telemedicine” OR “digital health”)) AND (TITLE-ABS-KEY(“self-care”)) AND (TITLE-ABS-KEY(“heart failure” OR “heart failure, diastolic” OR “heart failure, systolic”))
Search	Google Scholar
1	allintitle:telenursing AND self-care AND heart failure
Search	Web of Science
1	TS = (“telenursing” OR “nursing care” OR “patient participation” OR “telemedicine” OR “digital health”)
2	TS = (“self-care”)
3	TS = (“heart failure” OR “heart failure, diastolic” OR “heart failure, systolic”)
4	TS = (“telenursing” OR “nursing care” OR “patient participation” OR “telemedicine” OR “digital health”) AND TS = (“self-care”) AND TS = (“heart failure” OR “heart failure, diastolic” OR “heart failure, systolic”)
Search	ClinicalTrials.gov
1	Heart failure AND Telenursing

**Table 2:** Results of the methodological quality assessment of the included manuscripts.

JBI Critical Appraisal Tool for Randomized Controlled Trials		
Study	Score	Level
Choi, E.-Y. et al., 2023 [37]	9/13 (69%)	Low
Kitsiou, S. et al., 2025 [38]	12/13 (92%	High
Schmaderer, M. et al., 2022 [39]	9/13 (69%)	Low
Shara, N. et al., 2022 [40]	11/13 (85%)	High
Yanicelli, L. et al., 2021 [41]	9/13 (69%)	Low
Yokota, T. et al., 2023 [42]	9/13 (69%)	Low
JBI Critical appraisal tool of Quasi-experimental studies		
Study	Score	Level
Arslanian-Engoren, C. et al., 2021 [43]	7/9 (78%)	Medium
Kaneko, T. et al., 2025 [44]	7/9 (78%)	Medium
Longhini, J. et al., 2023 [45]	6/9 (67%)	Low
Marier-Tétrault, E. et al., 2024 [46]	8/9 (90%)	High
Purcell, C. et al., 2023 [47]	7/9 (78%)	Medium
Son, J-Y. et al., 2025 [48]	8/9 (90%)	High
Son, Y-J. et al., 2022 [49]	7/9 (78%)	Medium
JBI Critical appraisal tool of cohort studies		
Study	Score	Level
Knoll, K. et al., 2023 [50]	8/11 (73%)	Medium
JBI Critical appraisal tool of analytical cross-sectional studies		
Study	Score	Level
Ekola, T. et al., 2024 [51]	5/8 (63%)	Low

**Table 3:** Overall description of the studies included (*n* = 15).

Author/Year/Country	Aim	Study Design	Sample Size/Age	Measures and Instruments	Intervention	Key Findings
Arslanian-Engoren et al., (2021) [43] Michigan	To assess the feasibility and acceptability of a nurse-led, virtual, home-based cognitive training and self-care education intervention.	Quasi-Experimental Study	12 patients: 58.3% women; median age: 62.5 years; Average years of education: 17	TICS-M; SCHFI V 6.2. Patient characteristics were self-reported; app characteristics were assessed using a 5-point Likert-type scale. The computer interface evaluation included yes/no answers.	CASE-CCT, 10 sessions. Data were collected at baseline (T1), after the 10 sessions (T2), and at 2-month follow-up (T3). CCT: Patients navigated increasingly complex hallways and alleys to reach level-specific goals. Educational sessions.	Adequate SC scores increased from T1 to T2 across all subscales. Median SCHFI scores improved significantly in maintenance (T1–T2), management (T2–T3), and confidence (T1–T3).
Choi et al., (2023) [37] Korea	To develop a self-management mobile app for HF patients and evaluate its impact on physical, psychosocial, and behavioral outcomes.	Randomized controlled trial	Intervention (*n* = 36): age 70.3 (years), 52.8% male, 50% ≤ middle school, 7.9 years since diagnosis; Control (*n* = 34): age 79.4 (years), 42.1% male, 68.4% ≤ middle school, 7.4 years since diagnosis	NYHA classification; Geriatric Depression Scale; QoL (MacNew 40-item); Hill Bone 8-item; EHFScB 12-item.	Data were collected at baseline and 3 months. The app, guided by cardiac nurses, offered self-management content, diary monitoring, reminders, and chat support; controls received usual outpatient care.	The intervention improved NYHA class but not psychosocial or behavioral outcomes. The expert-developed app supports flexible patient engagement and access to health information without limitation of distance.
Ekola et al., (2024) [51] Finland	To assess the feasibility of a noninvasive telemonitoring system for use by HF patients and nurses in a pilot program.	Analytical cross-sectional study Mixed methods design	37 participants: 29 patients and 8 nurses. Nurses’ experience (years): 14.4; Patients’ mean age (years): 61,4; 75.9% men; 42.9% had HF for <2 years; 96.6% used medication; 89.7% used digital devices	Quantitative data were collected using a study-specific questionnaire. Qualitative data were obtained through semi-structured face-to-face focus group interviews.	Data were collected once. Using an Android tablet with the app and a digital scale, patients completed daily questionnaires. Data synced to the cloud with automatic feedback, while nurses intervened as needed.	Facilitators: Easy to use, improves SC, facilitates communication between nurses and patients, increases the feeling of safety. Barriers: Connectivity, patient and equipment burden, workload of nurses, protocol gaps, system challenges.
Kaneko et al., (2025) [44] Japan	To evaluate the impact of a home-based remote management program, utilizing a telemonitoring and telecoaching system, on HF and executive functions.	Quasi-Experimental Study	41 patients: 68% male sex; mean age, 64 years; 73% had a history of HF hospitalization; median number of HF hospitalizations, 2	Trail Making Test; Mini-Mental State Examination; NYHA classification.	Data were collected at baseline and 12 months. Home telemonitoring included self-care education and device training. Nurses monitored data five days/week and notified providers.	NYHA class improved, and Trail Making Test scores increased. Hospitalization rates were higher in patients with executive dysfunction than in those who were cognitively intact.
Kitsiou et al., (2025) [38] United States of America	To assess the effectiveness of a patient-centered mHealth program with personalized SMS to improve HF self-care.	Randomized Controlled Trial	27 patients: 11 in the intervention group and 14 in the control group; patients’ mean age: 56 years; 44% female; 76% NYHA class II and II	SCHFI version 7.2.; Self-Care Self-Efficacy; Dutch Heart Failure Knowledge Scale; KCCQ-12 short version	Patients were followed for 8 weeks. The mHealth program used 3 apps to collect data and send tailored SMS. The primary outcome was HF self-care; secondary outcomes included behaviors, health status, and adherence.	The intervention group improved in SCHFI scores, especially in self-efficacy and adherence, with fewer hospitalizations and the same number of ED visits. Patients found the program engaging and informative.
Knoll et al., (2023) [50] Germany	To assess the effectiveness of a combined telemonitoring and telecoaching program in recently hospitalized HF patients at high risk of re-hospitalization.	Retrospective case–control study	6065 HF patients recently hospitalized and at high risk for re-hospitalization; age > 40 years old	EHFScBs-9. Hospitalization risk was estimated using adaptable and comprehensive risk assessment methodology. Adherence was the proportion of expected telemonitoring data received.	Daily remote monitoring of HF and individualized telecoaching by trained nurses took place over 12 months. Alerts were reviewed, and the nurses interviewed patients by structured telephone contacts to assess and provide instructions.	Combined telemonitoring and telecoaching in high-risk HF patients reduced mortality and HF hospitalizations, with high adherence across ages. Telehealth is particularly valuable when in-person care is limited.
Longhini et al., (2023) [45] Italy	To assess the feasibility and acceptability of a nurse-led telemonitoring model for stable HF patients and their caregivers in primary care.	Quasi-experimental study	25 patients and 8 caregivers Median age (years): 72 for patients and 50.5 for caregivers 10 participants accepted using the app (6 were caregivers)	SCHFI 7.2 Italian version; CC-SCHFI Italian version; Qualitative semi-structured face-to-face interviews	Nurses supported participants via a mobile app or paper materials, providing education, motivational guidance, and regular follow-ups. Patients’ data were monitored daily. The self-care questionnaires were assessed over 3 months.	Facilitators: guidance, easy access, feeling heard, health awareness improvement. Barriers: distance, low digital skills, time, preference for paper diaries. Overall, the intervention enhanced SC, and encouraged management of HF.
Marier-Tétrault et al., (2024) [46] Canada	To evaluate the feasibility of implementing an innovative digital solution in a healthcare setting.	Quasi-Experimental Study	52 patients: 32 in the RPM+ and DTx+ group, 9 in the RPM− and DTx+ group, 11 in the RPM− and DTx− group; 71% had a mobile phone; 86% able to use the app; 75% NYHA class II, and half had LVEF < 40%	Quality of life was measured with the KCCQ-12; hospitalizations and HF medications were recorded at each visit.	Evaluation made at baseline, 6 weeks, and 12 weeks. The RPM+ group used the app to record vitals, weight, symptoms, to access educational content and receive medication suggestions. Nurses monitored daily and contacted patients.	Hospitalization occurred in 6% of RPM+ vs. 35% in RPM–. HF therapy optimization and QoL improved. For nurses, the major barrier was the high volume of incoming data, and for patients were low level of education and frailty.
Purcell et al., (2023) [47] United Kingdom	To evaluate how REACH-HF is being put into practice in regular healthcare settings within Scotland National Health Service.	Quasi-experimental study	101 patients and 26 caregivers Patients’ mean age (years): 68; 72% men; 94% NYHA class II and III Caregivers’ mean age (years): 62; 76% women; 65% were the partner	Minnesota Living with HF Questionnaire; EuroQoL-5; Modified PROM-CR+; SCHFI; Hospital Anxiety and Depression Scale; Health Literacy Questionnaire; Chromogranin-B HF	Data were collected at baseline and at 4 months in a 3-month program. Patients averaged 5 facilitator sessions (4 h 50 m) and received The HF Manual, exercise and stress management plans, a progress tracker, and family support materials.	REACH-HF improved health-related quality of life in HF patients, and showed limited caregiver benefits. COVID-19 adaptations did not reduce effectiveness, though hybrid delivery may be preferred.
Schmaderer et al., (2022) [39] United States of America	To evaluate the impact of mHealth self-management on symptoms and health-related quality of life	Randomized controlled trial	74 patients: 26 in the usual care group, 23 in the mHealth, and 25 in the mHealth+ Mean age 53.6 years; 54% female; 50% with high school or lower	HF Symptom Survey; EuroQol	Data were collected at baseline and at 3 months. Usual care group: recorded medication and weight; mHealth group: provided with additional app features; mHealth+ group: added nurse virtual visits focused on problem-solving and decision-making.	Shortness of breath drives HF care. Both mHealth groups improved; mHealth+ had fewer hospitalizations, ED visits, and stronger symptom effects. Nurse-virtual intervention may promote self-management and improve health care utilization outcomes.
Shara et al., (2022) [40] United States of America	To evaluate the feasibility of a voice-activated technology for the follow-up of HF patients, as well as its impact on clinical outcomes and health care utilization.	Randomized controlled trial	60 patients allocated 1:1 into two groups (the Alexa+ and the standard of care); Mean age: 54 years	Voice interface; Questionnaire; A tool developed to synthesize patients’ answers; Development of an alarm system.	A daily questionnaire assessed adherence to SC and HF medications and monitored symptoms of worsening over 3 months. Responses were summarized by a voice interface, providing personalized feedback.	A total of 77% agreed that the assistant aided in managing their health. Its use was linked to more HF hospitalizations and ED visits. Symptom severity varied, highlighting the need for a personalized approach.
Son et al., (2023) [49] Korea	To assess the initial effectiveness and usability of a new mobile phone-based self-care intervention for patients with HF.	Quasi-experimental study Mixed methods design	26 Korean HF patients Median age: 62 (years); 76.9% were male; median HF diagnosis: 2.6 (years); 42.3% had already received HF education; 19.2% had experience in the mHealth app use	Korean eHEALS; Korean Patient Knowledge Questionnaire; Korean EHFScBS-9; App quality assessed by the MARS.	Data were collected at baseline and 24 weeks on eHealth literacy, disease knowledge, and self-care. Phases: (1) literature review, (2) app development, (3) patient goal setting. Chatbot messages: 4/week for 8 weeks, then 3/week.	Scores for eHealth literacy, disease knowledge, and self-care behaviors improved significantly. The app improved awareness and motivation for routine self-care, highlighted challenges in adapting, and provided suggestions.
Son et al., (2025) [48] Korea	To test a novel mHealth intervention for patient–caregiver dyads to improve self-care adherence in HF.	Quasi-experimental study	70 dyads Patients’ mean age (years): 70.67; 64% were men Caregivers’ mean age (years): 59.34; 76% were women; 83% lived with the patient	EHFScBS-9, EuroQol, Brief Health Literacy Screener, Patient Knowledge Questionnaire, Patient Health Questionnaire-9, ENRICHD Social Support Instrument, HF Caregiver questionnaire, MARS user version	Data were collected at 1, 3, and 6 months post-enrollment. The dyadic mHealth program included a symptom-tracking app and a chatbot sending fixed messages three times weekly for 24 weeks.	The intervention group showed better SC adherence, HRQOL, and caregiver contribution over 6 months. Health literacy improved at 1 month. No significant effects were found for depression, social support, or caregiver burden.
Yanicelli et al., (2021) [41] Argentina	To evaluate the effectiveness of a home telemonitoring system in enhancing self-care and treatment adherence among HF patients.	Randomized controlled trial	30 patients were assigned 1:1 to the control group or the intervention group Average age: 52 years; 80% male; 60% unemployed; 75% with no high school education; most were in NYHA class II/III	EHFScB; Morisky Modified Scale.	Over 3 months, the application collected health parameters, delivered education, and generated alerts on a daily basis if a high-risk situation was detected. It was used alongside medical consultations over a 90-day monitoring period.	SC and treatment adherence improved in the intervention group. Patients were satisfied with the system and valued active disease management. Home telemonitoring system use may enhance adherence and reduce re-hospitalizations.
Yokota et al., (2023) [42] Japan	To assess the feasibility of the self-care intervention using a new app; to evaluate whether this mobile app enhances patients’ self-care behavior.	Randomized controlled trial	24 outpatients with CHF Mean age: 60 years; 75% men; 58% with daily habits of self-monitoring; 46% had been hospitalized due to worsening HF within the previous 3 months.	EHFScB-12; HF Knowledge Scale; General Self-Efficacy Scale; SF-8 Health Survey	Data were collected at baseline and at 2 months. The app offered self-monitoring, education, and automated HF alerts. The intervention group used the app, while the control group received an HF diary.	The intervention group demonstrated excellent adherence. Self-care behavior scores improved significantly, although HF knowledge, self-efficacy, and QoL scores showed no differences.

Legend: CASE-CCT = Cognitive Assessment, tailored Self-care Education and Computerized Cognitive Training; CC-SCHFI = Caregiver Contribution to Self-Care of Heart Failure Index; CCT = computerized Cognitive Training; CHF = chronic heart failure; DTx = digital therapeutics; eHEALS = eHealth Literacy Scale; EHFScB/EHFScBS = European Heart Failure Self-Care Behavior Scale; HF = heart failure; HRQOL/QoL = Health-Related Quality of Life/Quality of Life; KCCQ-12 = Kansas City Cardiomyopathy Question-naire-12; LVEF = Left Ventricular Ejection Fraction; MARS = Mobile Application Rating Scale; NYHA = New York Heart Association; PROM-CR = Patient-Reported Out-come Measure-Cardiac Rehabilitation; REACH-HF = Rehabilitation EnAblement in CHronic Heart Failure; RPM = remote patient monitor-ing; SC = self-care; SCHFI = Self-Care Heart Failure Index; SF-8 = Short Form-8; TICS-M = Telephone Interview of Cognitive Status.

**Table 4:** Effects of telenursing on the self-care and healthcare-related outcomes in CHF.

Study and Design	Sample Size and Follow-Up	Outcome Domain	Effect Estimate	Precision, Confidence Intervals, and *p*-Values
Arslanian-Engoren et al. [43] Quasi-experimental Study	Sample size: 12 participants | Follow-up: 30 days	Self-Care, Cognition, Healthcare Utilization	Self-Care Medians: Maintenance increased from 71 to 92; management increased from 70 to 85; confidence increased from 47 to 81 | Readmissions: 25 percent (2 out of 8 participants) readmitted	Maintenance: *p*-value equal to 0.01; Management: *p*-value equal to 0.045; Confidence: *p*-value equal to 0.010
Choi et al. [37] Randomized Controlled Trial	Sample size: 70 participants (Intervention Group: 36, Control Group: 34) | Follow-up: 12 weeks	Physiological Factors, Self-Care Behaviors, Psychosocial Outcomes	Functional Class Difference: Intervention Group −0.32 versus Control Group −0.28 | Physiological Ratio Difference: Intervention Group −0.89 versus Control Group +1.72 | Self-Care Management Difference: Intervention Group +6.97 versus Control Group +2.96	Functional Class: *p*-value equal to 0.003; Physiological Ratio: *p*-value equal to 0.024 | Self-Care Management: *p*-value greater than 0.05
Kaneko et al. [44] Quasi-experimental Study	Sample size: 41 participants | Follow-up: 12 months	Cognition, Functional Status, Clinical Events	Hospital Readmission Risk: Adjusted Hazard Ratio equal to 4.97 (Subgroup with higher cognitive function: Hazard Ratio equal to 6.97) | Mortality Risk: Adjusted Hazard Ratio equal to 1.93	Readmission Hazard Ratio with 95 percent Confidence Interval between 1.15 and 21.4; *p*-value equal to 0.031 | Mortality Hazard Ratio with 95 percent Confidence Interval between 0.27 and 13.9; *p*-value equal to 0.512
Kitsiou et al. [38] Randomized Controlled Trial	Sample size: 27 participants (Intervention Group: 11, Control Group: 14) | Follow-up: 30 days	Self-Care, Psychosocial Outcomes	Effect Sizes (Cohen’s d): Maintenance d equal to 0.19; Management d equal to 0.25 | Self-Efficacy d equal to 0.68; Health Beliefs d equal to 0.94	Maintenance with 95 percent Confidence Interval between −0.65 and 1.02 | Self-Efficacy with 95 percent Confidence Interval between 0.03 and 1.33
Knoll et al. [50] Retrospective case–control study	Sample size: 6065 high-risk participants | Follow-up: 1 and 2 years	Clinical Outcomes, Self-Care Behaviors	Intention-to-Treat Mortality: Hazard Ratio equal to 0.62 | 1-Year Mortality: Telehealth 5.8 percent versus Usual Care 11.0 percent | Per-Protocol Mortality: Hazard Ratio equal to 0.47	Intention-to-Treat Hazard Ratio with 95 percent Confidence Interval between 0.56 and 0.69; *p*-value less than 0.001 | Per-Protocol Hazard Ratio with 95 percent Confidence Interval between 0.42 and 0.53; *p*-value less than 0.001
Longhini et al. [45] Quasi-experimental Study	Sample size: 33 participants (25 patients and 8 caregivers) | Follow-up: 3 months	Dyadic Self-Care	Patient Median Difference: Maintenance +3.0; Monitoring +9.1; Management +7.5 | Caregiver Median Difference: Maintenance +9.0; Monitoring +6.4	Patient Maintenance: *p*-value equal to 0.007; Monitoring: *p*-value less than 0.001; Management: *p*-value less than 0.001
Marier-Tétrault et al. [46] Quasi-experimental Study	Sample size: 52 participants (32 with Active Remote Monitoring, 20 with Passive Remote Monitoring) | Follow-up: 12 weeks	Clinical Events, Quality of Life, Feasibility and Adherence	All-Cause Readmissions: Active Monitoring 6 percent versus Passive 35 percent | Heart Failure Readmissions: Active Monitoring 6 percent versus Passive 25 percent | Quality of Life Difference: +10.6 points in adherent users	Descriptive metrics | *p*-values and 95 percent Confidence Interval not reported
Purcell et al. [47] Quasi-experimental Study	Sample size: 127 participants (101 patients and 26 caregivers) | Follow-up: 4 months	Quality of Life, Self-Care, Psychosocial Outcomes, Economic Outcomes	Mean Quality of Life Difference: −9.8 points | Clinically Meaningful Improvement: 63 percent of patients | Self-Care Maintenance Difference: +7.4 points	Primary Quality of Life with 95 percent Confidence Interval between −13.2 and −6.4; *p*-value less than 0.001 | Self-Care Maintenance with 95 percent Confidence Interval between 4.7 and 10.2; *p*-value less than 0.0001 | Average cost: 397.22 British Pounds per patient
Schmaderer et al. [39] Randomized Controlled Trial	Sample size: 74 participants (26 Usual Care, 23 Simple Mobile Health, 25 Advanced Mobile Health) | Follow-up: 3 months	Clinical Events, Healthcare Utilization, Symptoms and Quality of Life	Readmission Rate: Advanced Mobile Health 16 percent versus Simple Mobile Health 56 percent versus Usual Care 50 percent | Quality of Life (Advanced Mobile Health versus Usual Care): Cohen’s d equal to 0.32	Readmissions (Advanced Mobile Health versus Usual Care): Fisher’s Exact Test equal to 0.017 with *p*-value equal to 0.02 | Quality of Life between groups: *p*-value greater than 0.05
Shara et al. [40] Randomized Controlled Trial	Sample size: 60 participants (30 with Advanced Alexa Voice Assistant, 30 Control Group) | Follow-up: Average of 87 days	Feasibility and Technology Utilization, Healthcare Utilization	Heart Failure Contact Rate (Per-Protocol): Voice Assistant Group 20 percent versus Control Group 0 percent | Active Participation: 66.7 percent retention	Overall Heart Failure Contacts: Fisher’s Exact Test with *p*-value equal to 0.021
Son et al. [49] Quasi-experimental Study	Sample size: 26 participants | Follow-up: 24 weeks	Self-Care Behaviors, Digital Health Literacy, Disease Knowledge, Feasibility	Pre- and Post-Intervention Median Difference: Digital Literacy +3.2; Knowledge +2.0; Self-Care Behaviors +10.0 | Correlation: Digital Literacy versus Self-Care r equal to 0.40	Digital Literacy: *p*-value equal to 0.005; Knowledge: *p*-value less than 0.001; Self-Care: *p*-value less than 0.001
Son et al. [48] Quasi-experimental Study	Sample size: 70 patient–caregiver dyads (35 Intervention Group, 35 Control Group) | Follow-up: 1, 3, and 6 months	Dyadic Self-Care, Quality of Life, Digital Health Literacy and Knowledge	Patient Self-Care (Generalized Estimating Equation Beta Coefficient at 6 months): 4.68 | Caregiver Contribution at 6 months: Beta Coefficient equal to 8.76 | Patient Quality of Life at 6 months: Beta Coefficient equal to 0.07	Patient Self-Care at 6 months with 95 percent Confidence Interval between 0.99 and 8.37; *p*-value equal to 0.01 | Caregiver Contribution with 95 percent Confidence Interval between 4.63 and 12.88; *p*-value less than 0.001 | Patient Quality of Life with 95 percent Confidence Interval between 0.00 and 0.13; *p*-value equal to 0.049
Yanicelli et al. [41] Randomized Controlled Trial	Sample size: 30 participants (15 Intervention Group, 15 Control Group)	Medication Adherence, Self-Care Behaviors, Clinical Events	Mean Difference in Self-Care Score: Intervention Group +11.48 versus Control Group −9.11 | Clinical Events: 2 hospitalizations prevented by weight alerts	Self-Care Difference between Groups (Student’s *t*-Test): *p*-value equal to 0.004 | Medication Adherence between Groups: *p*-value equal to 0.8
Yokota et al. [42] Randomized Controlled Trial	Sample size: 24 participants (13 Intervention Group, 11 Control Group) | Follow-up: 2 months	Self-Care Behaviors, Vital Signs Monitoring, Physical Activity, Salt Intake, Clinical Events	Intervention Group achieved significantly better self-care behavior scores at 2 months	Self-Care Behavior Score between Groups: *p*-value less than 0.05 | Step Count Difference between Groups: *p*-value equal to 0.06

## Data Availability

No new data were created or analyzed in this study. Data sharing is not applicable to this article.

## Supplementary Materials

The following supporting information can be downloaded at: https://www.mdpi.com/article/10.3390/clinpract16100183/s1, PRISMA 2020 checklist.
